# Coordinated regulation of Myc trans-activation targets by Polycomb and the Trithorax group protein Ash1

**DOI:** 10.1186/1471-2199-8-40

**Published:** 2007-05-22

**Authors:** Julie M Goodliffe, Michael D Cole, Eric Wieschaus

**Affiliations:** 1 Department of Molecular Biology, Princeton University, Princeton, NJ 08544, USA; 2 Department of Biology, University of North Carolina Charlotte, 9201 University City Blvd., Charlotte, NC 28223, USA; 3 Departments of Pharmacology and Genetics, HB 7936, Dartmouth Medical School, One Medical Center Drive, Lebanon, NH 03756, USA

## Abstract

**Background:**

The Myc oncoprotein is a transcriptional regulator whose function is essential for normal development. Myc is capable of binding to 10% of the mammalian genome, and it is unclear how a developing embryo controls the DNA binding of its abundant Myc proteins in order to avoid Myc's potential for inducing tumorigenesis.

**Results:**

To identify chromatin binding proteins with a potential role in controlling Myc activity, we established a genetic assay for dMyc activity in *Drosophila*. We conducted a genome-wide screen using this assay, and identified the Trithorax Group protein Ash1 as a modifier of dMyc activity. Ash1 is a histone methyltransferase known for its role in opposing repression by Polycomb. Using RNAi in the embryo and Affymetrix microarrays, we show that *ash1 *RNAi causes the increased expression of many genes, suggesting that it is directly or indirectly required for repression in the embryo, in contrast to its known role in maintenance of activation. Many of these genes also respond similarly upon depletion of *Pc *and *pho *transcripts, as determined by concurrent microarray analysis of *Pc *and *pho *RNAi embryos, suggesting that the three are required for low levels of expression of a common set of targets. Further, many of these overlapping targets are also activated by Myc overexpression. We identify a second group of genes whose expression in the embryo requires Ash1, consistent with its previously established role in maintenance of activation. We find that this second group of Ash1 targets overlaps those activated by Myc and that ectopic Myc overcomes their requirement for Ash1.

**Conclusion:**

Genetic, genomic and chromatin immunoprecipitation data suggest a model in which Pc, Ash1 and Pho are required to maintain a low level of expression of embryonic targets of activation by Myc, and that this occurs, directly or indirectly, by a combination of disparate chromatin modifications.

## Background

Cancer can arise in many ways, one of the most potent being deregulation of the *myc *proto-oncogene. Myc is a transcription factor, and its C-terminal domain is required for dimerization with its partner Max and its subsequent binding to DNA. It also possesses an N-terminal trans-activation domain that is required for both Myc's biological activities of transcriptional activation and repression. Activation by Myc involves binding to the nuclear protein TRRAP, which recruits histone acetyltransferase complexes to Myc activation targets. Subsets of target genes include those that mediate cell cycle progression, cell growth, genomic instability, angiogenesis, and inhibit differentiation [1-3]. Myc also acts as a repressor of a different set of targets, in addition to interfering with the function of activators such as Miz1 [4,5]. Myc binding to DNA is dependent on chromatin context, such that Myc sites having certain chromatin modifications are almost always bound, and Myc sites lacking those certain modifications are rarely bound by Myc [6].

Experiments in mammalian systems have answered many questions regarding Myc biology, though their limitations in broad based discovery led us to investigate Myc biology in *Drosophila*. The dMyc protein is highly similar to c-Myc in the residues required for DNA binding, and the gene has a similar exon and intron structure [7,8]. dMyc can functionally replace c-Myc in transformation assays with activated Ras and in rescue experiments with mouse cells null for c-*myc *[8,9]. In Drosophila, dMyc regulates RNA Pol I production of ribosomal RNAs, is required for oogenesis and larval growth, and overexpression of *dmyc *in clones of the *Drosophila *imaginal disc confers a growth advantage to cells having greater amounts of *dmyc *expression than their neighbors [10-14].

In a transgenic mouse model using induction of Myc in hepatocytes, the ability of Myc to induce proliferation and tumorigenesis was shown to vary greatly depending on the age of the mouse at the time Myc was induced, suggesting that the differentiation states of cells can mediate the response to Myc [15]. In *Drosophila*, differentiated or undifferentiated cell states are maintained by the Polycomb Group (PcG) and Trithorax Group (TrxG) of proteins, which are thought to bind to repressed or activated loci, and thus to stabilize patterns of gene expression [16,17]. We reported previously that repression by Myc in *Drosophila *requires Polycomb, a chromatin-binding transcriptional repressor [18]. Previously characterized phenotypes manifested in PcG mutants reflect a loss of repression of homeotic genes, and the members of the PcG group were additionally defined by their ability to enhance each other's dominant phenotypes [19,20] (and references within). Conversely, mutant phenotypes of members of the TrxG reflect a loss of *activation *of homeotic genes, and these mutations behave as suppressors of mutations in Pc. Many of the members of the PcG of proteins have been found in two separate protein complexes, PRC1 and PRC2, neither of which bind to DNA alone. PRC1 includes Pc, Ph, Psc and dRing1; PRC2 includes Esc; a histone methyltransferase, E(z); and Su(z)12. PcG proteins bind to hundreds of sites in the genomes of mammals and *Drosophila *[21-25], and repression of homeotic genes mediated by PcG proteins is experimentally linked to cis regulatory elements known as PREs (Polycomb Response Elements, reviewed in [26,27]). The PcG proteins Pho and dSfmbt bind to sites within PREs, recruiting PRC2, followed by recruitment of PRC1 [28-32]. TrxG proteins bind to over 100 sites on polytene chromosomes and can be found in nucleosome remodeling complexes such as the BAP complex (Brahma Associated Protein complex: Brahma, Moira, Osa and Snr1). Trx and Ash1 (absent, small or homeotic discs 1) are SET domain histone methyltransferases (reviewed in [33]).

Repression by Myc requires Pc for many targets, which includes the *dmyc *locus itself [18]. In the present study, we describe a novel genetic assay that we use to find additional *Drosophila *gene products involved in repression of the *dmyc *gene. Using this assay, we screened the *Drosophila *genome for modifiers of *dmyc *expression. We then used RNAi to deplete levels of our genetic modifiers, Ash1 and Pho, in the embryo and used Affymetrix microarrays to analyze gene expression changes in these embryos with or without ectopic Myc. Our genetic and genomic data allow us to describe the role of Pho and Ash1 in Myc-mediated repression and in control of Myc trans-activation targets in the embryo. These data provide the first evidence for a functional link between canonical Pc and Trx Group proteins.

## Results

### *Pho *and *Psc *are involved in repression of *dmyc*

**Figure 1:**
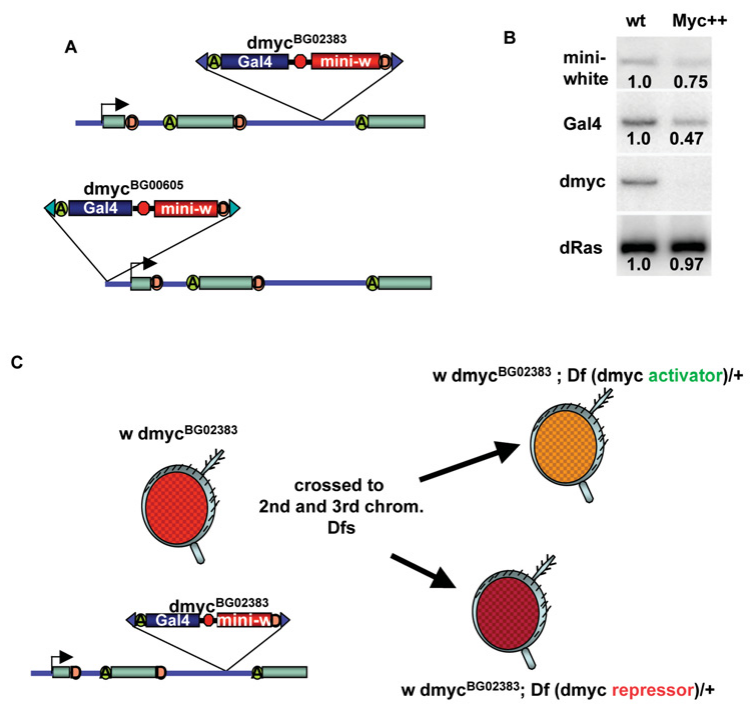
**A P element insertion in the *dmyc *locus is a reporter for *dmyc *activity**. (A) A diagram indicating the exons (green rectangles) and introns (thick blue line) of the *dmyc *gene on the X chromosome. A pGT1 insertion in the second intron (*dmyc*^*BG*02383^, top) inserts the *Gal4 *(blue rectangle) and *mini-white *(red rectangle) reporters. A second insertion upstream of the *dmyc *transcription site is also shown (*dmyc*^*BG*00605^, bottom) (B) *Gal4 *and *mini-white *reporters exhibit *dmyc *autorepression. RT-PCR amplification products are shown from embryos with *dmyc*^*BG*02383 ^having *Gal4 *or *Gal4*; *UAS-dmyc *transgenes, and therefore wild type for Myc (wt) or producing ectopic Myc (Myc++), respectively. The numbers under each band indicate the relative band intensities between pairs as indicated by phosphorimaging. (C) Scheme for genetic screen for modifiers of *dmyc *activity. Females homozygous for *dmyc*^*BG*02383 ^on the X Chromosome were crossed to males of the Second and Third Chromosome deficiency "kits" (Bloomington). Fly heads are depicted in profile, cartooning the changes in eye color expected when a fly has both *dmyc*^*BG*02383 ^and either a mutation in a *dmyc *activator (upper right) or a mutation in a *dmyc *repressor (lower right).

Pc binds to methylated histone H3 at lysine 27 (H3K27), mediating repression of many genetic loci by Myc [18]. In an investigation of other PcG gene products potentially involved in this repression by Myc and Pc, we chose three candidates to test: Posterior sex combs (Psc), which is in a core repressive complex with Pc [34]; E(z), which is a histone methyltransferase that methylates histone H3 lysine 27 and recruits Pc repression [35-37]; and Pho, which is one of two DNA-binding proteins of the group and recruits E(z) [28,30-32]. Our strategy for testing Psc, E(z) and Pho involved a genetic test for modification of expression at the *dmyc *locus. Each of two different P element insertions in the *dmyc *locus, *dmyc*^*BG*02383 ^and *dmyc*^*BG*00605^, provides two reporters for *dmyc *expression: a promoterless yeast *Gal4 *gene that is expressed by the upstream *dmyc *promoter, and a *Drosophila *eye color *mini-white *gene whose expression is influenced by the regulation of the locus (Figure 1A) [38,39].

We first determined whether these reporters were sensitive to the regulation of the *dmyc *gene, using the phenomenon of *dmyc *autorepression. In this assay, ectopic *dmyc *leads to a reduction in the expression from the endogenous locus [18]. We obtained high levels of ectopic Myc in embryos with the *dmyc*^*BG*02383 ^allele (see Methods for genotypes and crosses). We collected RNA from *dmyc*^*BG*02383 ^embryos with or without ectopic *dmyc *and used RT-PCR to amplify transcripts from the *Gal4 *and *mini-white *reporters, the first exon of *dmyc *(which amplifies only the endogenous transcript and not the exogenous transcript), and a loading control, *dRas*. Expression of endogenous *dmyc *in embryos with the P-element insertion is normal, and Northern blotting indicates that the mature transcript size is the same as in wild type (Figure 1B and data not shown). Endogenous *dmyc *expression detected by RT-PCR in embryos with ectopic Myc was close to zero (Figure 1B), indicating that autorepression is intact in *dmyc*^*BG*02383 ^embryos. Phosphorimaging data revealed a reproducible reduction in both *mini-white *and *Gal4 *expression in embryos with ectopic *dmyc *(Figure 1B). The effect was small but striking, given that the embryos contain sources of *mini-white *and *Gal4 *in addition to those from the insertion in *dmyc *(i.e. the two transgenes that we used to provide ectopic *dmyc*, *UAS dmyc *and *Gal4*, both have *mini*-*white *associated with them). These results provided the molecular validation for our genetic strategy, linking *dmyc *regulation to the expression of the reporters within the locus.

**Figure 2:**
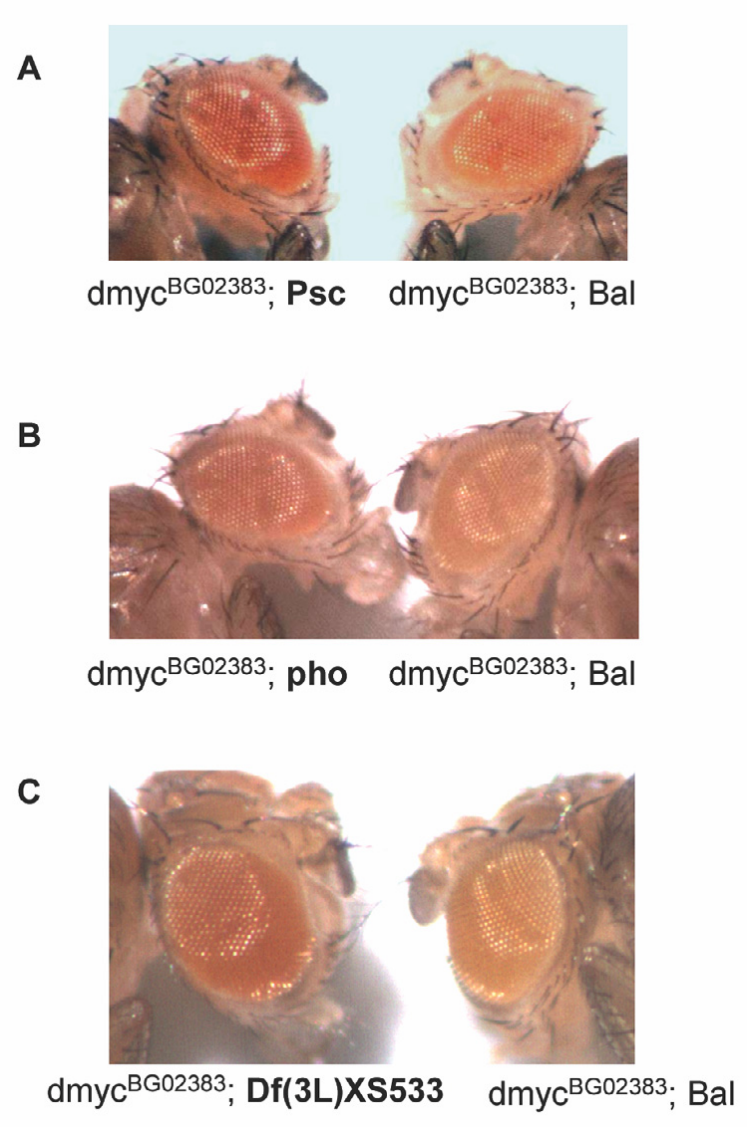
***Psc*,*pho *and *ash1 *behave as genetic repressors of the *dmyc *locus**. (A) Male siblings are shown, both hemizygous for *dmyc*^*BG*02383^, and either heterozygous for *Psc*^1 ^(left), or the balancer chromosome (*CyO*). (B) Male siblings are shown, both hemizygous for *dmyc*^*BG*02383^, and either heterozygous for *pho*^1 ^(left), or the balancer chromosome (*In(4)ciD*). (C) Male siblings are shown, same as in A and B, though heterozygous for either Df(3L)XS533, a Df that includes *ash1 *(left), or the balancer chromosome (*TM6B*).

We reasoned that reducing the dosage of a gene involved in *dmyc *repression might elevate the *mini-white *expression above that normally observed in *dmyc*^*BG*00605 ^and *dmyc*^*BG*02383 ^flies. Therefore, we generated flies with mutations in candidate *dmyc *repressors combined with the *dmyc *reporters and examined levels of *mini-white *expression (see Methods for genetic crosses and scoring procedures). Heterozygosity (i.e. one mutant chromosome and one wild type chromosome) for a *Pc *mutation did not increase *mini-white *levels of *dmyc*^*BG*02383 ^males, but did affect *mini-white *levels of *dmyc*^*BG*00605 ^(16% dark *Pc*^3 ^males versus 0 dark non-mutant control male siblings, Table 1). Heterozygosity for a *Psc *mutation affected both insertions in *dmyc*, increasing the *mini-white *levels compared to those of the non-mutant siblings in both cases (60% dark for *dmyc*^*BG*02383^, 44% dark for *dmyc*^*BG*00605^, 18% dark for the non-mutant controls, Figure 2A and Table 1). Males heterozygous for either of two different loss-of-function alleles of *E(z)*, *E(z)*^5 ^and *E(z)*^61^, did not increase *mini-white *levels of either insertion in *dmyc*; *E(z) *males and non-mutant male siblings had indistinguishable *mini-white *intensities. Males heterozygous for a hypomorphic mutation in *pho*, *pho*^1^, had greater numbers of males with dark eyes than their non-mutant siblings, with 57% of *pho*^1 ^heterozygotes having dark eyes compared to 5% of the non-mutant flies having dark eyes (Figure 2B and Table 1). Therefore, we considered *dmyc*^*BG*00605 ^to be the more sensitive reporter for *dmyc *expression because it revealed regulation by Pc. *Psc *and *pho *mutations affected both insertions, suggesting their involvement in Pc/Myc repression. However, neither insertion was affected by *E(z) *mutations, suggesting that *E(z) *may not be involved in repression by Myc or that mutations in *E(z) *are not haploinsufficient with respect to Myc activity.

**Table 1:** Deficiencies and mutants that behave as genetic repressors of *dmyc*

**Deficiency/mutant**	**Cytology**	**P value**
Df(2R)vg135	47F04-048A;049A-B, 49A-B;049D-E	1.50E-21
Df(3L)Aprt-32	62B01;062E03	2.73E-06
Df(3L)BSC13	66B12-C01;066D02-04	9.91E-04
Df(3L)lxd6	67E05-07;068C02-04	2.32E-07
Df(3L)BSC10	69D04-05;069F05-07	4.72E-206
Df(3L)fz-CAL5	70C02-06;070E01	2.34E-33
Df(3L)kto2	76B01-02;076D05	1.84E-52
Df(3L)XS533	76B04;077B	1.60E-21
Df(3R)p712	84D04-06;085B06, 025D;085B06	5.03E-10
Df(3R)M-Kx1	86C01;087B01-05	1.93E-05
Df(3R)T-32	86E02-04;087C06-07	4.02E-95
ash1 RE418	76B8-9	4.09E-58
Pc3*	78C6-7	5.65E-10
Psc1	49E6	8.51E-57
pho1	102D6	2.87E-34
E(z)	67E5	no change
ash2	96A13	no change

P values were computed using a chi-square test statistic. The asterisk refers to the combination of *Pc*^3 ^and *dmyc*^*BG*00605^, rather than *dmyc*^*BG*02383^.

### Pho participates with Pc in repression of Myc targets

**Figure 3:**
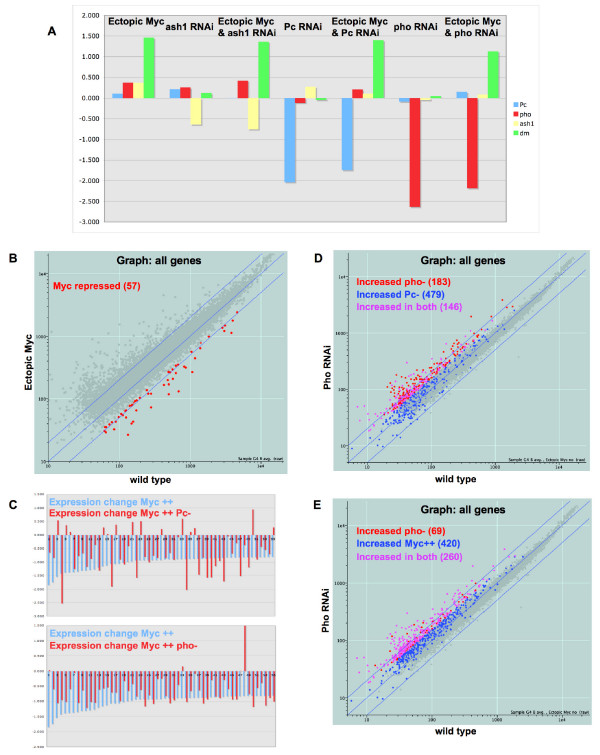
**Pho behaves similarly to and differently from Pc with respect to Myc activity**. For all scatter-plot graphs, the diagonal blue lines indicate zero change and 2-fold changes in either direction. Our thresholds for labeling genes as increasing or decreasing are not based on fold changes (see Methods), and therefore the locations of the colored dots may or may not correspond to a 2-fold change in expression. (A) Results of genetic manipulations. The log_2 _ratios of normalized intensities are graphed for each manipulated gene in each manipulated embryo. The left-most set of bars are from embryos expressing ectopic dmyc, and each bar shows the change in *Pc*, *pho*, *ash1 *and *dmyc *levels, respectively, in those embryos compared to embryos with just Gal4 (log_2 _(intensity Gal4 dmyc/intensity Gal4)). The next set of bars shows the changes in *Pc*, *pho*, *ash1 *and *dmyc *in embryos with *ash1 *RNAi, and so on. The genetic manipulation is indicated above each set of 4 bars. (B) Expression levels of 8865 genes are plotted by their basal levels along the X axis and their levels upon expression of ectopic Myc along the Y axis. Genes repressed by Myc are shown in red, n = 57. (C) Pc and Pho are required for Myc repression of half its targets. Expression changes, represented as log_2 _of ratios of expression over basal, of 55 Myc repressed transcripts are shown with changes with ectopic Myc (blue bar) next to their expression changes with ectopic Myc and *Pc *RNAi combined (red bar, top), or ectopic Myc and *pho *RNAi combined (red bar, bottom). The same 55 genes are graphed in the same order in both the upper and lower panels. (D) Expression levels of 8865 genes are plotted by their basal levels along the X axis and their levels upon *pho *RNAi along the Y axis. Red dots indicate genes whose levels rise with *pho *RNAi alone, blue dots indicate genes whose levels rise with *Pc *RNAi alone, and purple dots indicate genes whose levels rise with each of *Pc *or *pho *RNAi. (E) Expression levels of 8865 genes as in D, showing changes with ectopic Myc. Blue dots indicate genes whose levels rise with ectopic Myc. Red dots indicate genes whose levels rise with *pho *RNAi, purple indicates genes whose levels rise in each of ectopic Myc and *pho *RNAi.

**Figure 4:**
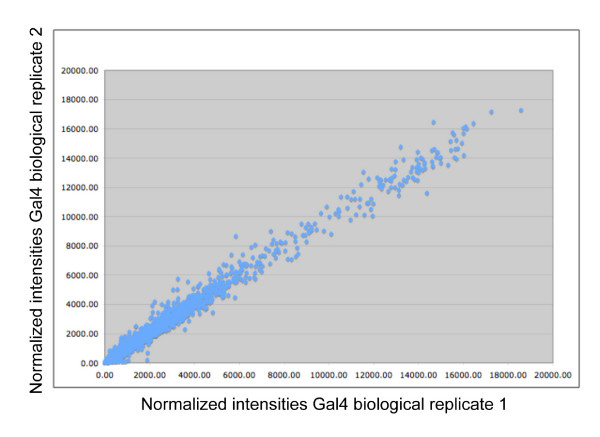
Biological replicates of microarray hybidizations are highly comparable. Normalized intensities for all 18660 transcripts on the Affymetrix Drosophila Genome 2.0 array are graphed for one replicate on the X axis, and the second replicate is the Y axis. Both sets were normalized to the average signal for the chip, and to control intensities.

The Pho protein is thought to recruit Pc to PREs [30-32] and in that way facilitates Pc's repressive activity. Requirements for Pho activity might therefore be expected to parallel those for Pc with respect to repression by Myc, as suggested by our *mini-white *assay described above. To examine this possibility, we injected embryos with dsRNA directed at *pho*, reducing transcript levels 6-fold (Figure 3A). We also induced RNAi for *pho *in embryos with ectopic Myc to examine the role that Pho has in Myc function. In addition to *pho *RNAi, we repeated the *Pc *RNAi experiment (4-fold reduction in *Pc *levels) for comparison using the Drosophila Genome 2.0 Affymetrix arrays (Figure 3A). As described previously [18], we also injected embryos with buffer for our controls, injected 200 embryos for each array hybridization, and duplicated the entire experiment, for a total of 400 injected embryos analyzed per genetic condition. We injected pre-cellularization embryos, and allowed them to age for 20 hours at 18°C before isolating RNA. This technique of using RNAi rather than known mutations in *pho *and *Pc *for our microarray analysis has several advantages. First, variability between genetic backgrounds is eliminated, since genetically identical mothers provided the embryos for our experiments from array to array, no matter what gene we depleted by RNAi or whether the fathers provided ectopic Myc or not (Figure 4). Second, transgene expression is constant from chip to chip because we used the identical Gal4 source and *UAS-dmyc *for all experiments, without concern for the logistics of combining three different genetic situations in one set of embryos. Third, because we reduced both maternal and zygotic transcripts by RNAi, our experiments did not require that we generate germ line clones to eliminate the maternal contribution of transcripts.

Our experiments reveal 329 target genes that appear to be repressed by Pho in normal embryos, since their levels increase following *pho *RNAi (see Methods for list generation). A larger number of Pc repression targets were identified following *Pc *RNAi (625, Table 2), and consistent with the view that Pc and Pho work together, the two groups of targets show substantial overlap; 146 of the 329 pho targets are also Pc targets (Figure 3D). That the remaining 183 Pho targets do not appear to overlap Pc targets may reflect the limitations of the threshold techniques used to identify targets in our experiments. It may also reflect differences of specific targets in their sensitivity to reductions in Pc and Pho (see Discussion). The shared regulatory roles of Pc and Pho extend beyond normal development to those 57 genes that are repressed in embryos following ectopic Myc accumulation (Figure 3B). 26 of these are no longer repressed to the same degree in the absence of Pho, and 25 are no longer repressed to the same degree in the absence of Pc (in the combined condition of ectopic Myc and RNAi of *Pc *or *pho*, Figure 3C and Table 3). These genes that fail to be repressed in the absence of Pc or Pho do not overlap completely. 15 are affected by Pc and Pho similarly, 10 are affected by Pc and not Pho, and 10 are affected by Pho and not Pc. We conclude that normal levels Pho and Pc are required for Myc's repressive activity, though not necessarily at the same time.

**Table 2:** Numbers of genes responding to ectopic Myc, *ash1 *RNAi (*ash1*-), (*pho*-), *Pc *RNAi (*Pc*-), and Myc combined with each

**List**	**Number of genes**
Myc activated	680
Myc repressed	57
up ash1-	239
down ash1-	159
up pho-	329
down pho-	69
up Pc-	625
down Pc-	76
up Myc ash1-	675
down Myc ash1-	120
up Myc pho-	486
down Myc pho-	82
up Myc Pc-	462
down Myc Pc-	291

See Methods for list generation.

**Table 3:** Gene list matrix

	**Myc activated**	**Myc repressed**	**up ash1-**	**down ash1-**	**up pho-**	**down pho-**	**up Pc-**	**down Pc-**	**up Myc ash1**	**down Myc ash1-**	**up Myc pho-**	**down Myc pho**	**up Myc Pc-**	**down Myc Pc-**
**Myc activated**	680	0	142	70	260	4	210	9	504	4	373	0	146	21
**Myc repressed**	0	57	1	3	0	11	10	6	0	47	0	31	0	32
**up ash1-**	142	1	239	0	139	4	130	0	142	6	156	4	49	9
**down ash1-**	70	3	0	159	35	4	26	8	53	9	22	7	11	20
**up pho-**	260	0	139	35	329	0	146	6	238	1	207	0	61	16
**down pho-**	4	11	4	4	0	69	14	8	7	19	7	14	1	21
**up Pc-**	210	10	130	26	146	14	625	0	227	17	209	8	161	12
**down Pc-**	9	6	0	8	6	8	0	76	1	12	3	7	0	47
**up Myc ash1-**	504	0	142	53	238	7	227	1	675	0	401	0	198	17
**down Myc ash1-**	4	47	6	9	1	19	17	12	0	120	1	57	0	63
**up Myc pho-**	373	0	156	22	207	7	209	3	401	1	486	0	149	18
**down Myc pho-**	0	31	4	7	0	14	8	7	0	57	0	82	0	55
**up Myc Pc-**	146	0	49	11	61	1	161	0	198	0	149	0	462	0
**down Myc Pc-**	21	32	9	20	16	21	12	47	17	63	18	55	0	291

Genes in each list are shown for their membership in every other list.

**Figure 5:**
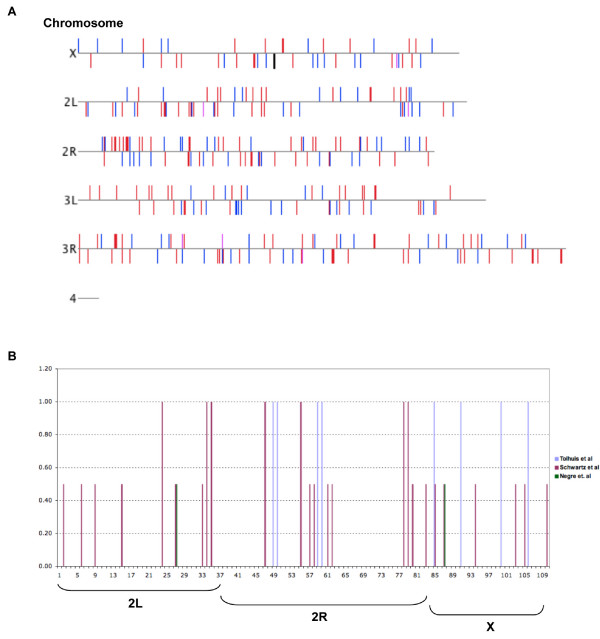
**Pc and Myc responsive genes are not clustered near genomic Pc binding sites**. (A) Chromosomes of the Drosophila genome are shown, with a vertical blue bar above or below (plus or minus strand, respectively) each chromosome to indicate Myc repressed genes. Red vertical bars show the locations of Pc repressed genes, and lilac vertical bars show genes repressed by both. (B) 110 Pc repressed genes on the 2^nd ^and X Chromosomes are graphed for their location overlapping or near a Pc binding site. According to data published by three different groups, a gene overlapping a Pc binding site is given a score of 1, a gene near a Pc binding site is given a score of 0.5, and a gene not near a Pc binding site is given a score of 0. Bars are labeled blue, B. Tolhuis et al., *Nat Genet *(Apr 20, 2006); magenta, Y. B. Schwartz et al., *Nat Genet *38, 700 (Jun, 2006); green, N. Negre et al., *PLoS Biol *4, e170 (Apr 20, 2006). We did not plot genes on the 3^rd ^Chromosome because the data set from Tolhuis et. al. does not include it.

We examined the possibility that Pc mediates its repressive effects upon dMyc targets by utilizing previously characterized Polycomb Responsive Elements (= PREs). The location of both Pc and Myc repressed genes, however, provides no evidence of close proximity to each other (Figure 5A). Because we were able to map target genes to chromosomes using Genespring with Affymetrix Drosophila Genome 1 data and not Genome 2.0, we used our previous set of Pc repressed genes to compare to the locations of likely PREs [18]. We compared the genomic locations of 214 Pc repressed genes to the genomic locations of experimentally determined Pc binding sites [24,25,40]. 13 of the 214 Pc responsive genes in our data overlap Pc binding sites, and 30 of the remaining 214 are within two cytological number divisions of Pc binding sites for one set of published data (i.e. gene at 61C7 and Pc binding at 61C9 or closer) [40]. Compared to a different set of published Pc binding sites, 8 of 214 Pc responsive genes of our set are within Pc binding sites [25]. The overlap is small, though interestingly, the overlap of the two previously published Pc binding sites with each other is also small: just 1 of 43 genes with Pc binding sites in our set is common to the data sets of both Schwartz et. al. and Tolhuis et. al. A third set of mapped Pc binding sites describes many fewer Pc binding sites, and two of those overlap our Pc responsive genes [24]; these two are also described as being Pc binding sites by Swartz et. al. (Figure 5B). We conclude that Pc binding is dynamic, with no absolute set of Pc binding sites. Each set of data is a snapshot of the current and changing position of Pc, and our gene expression data is consistent with the dynamic and possibly stochastic nature of Pc binding to the genome.

Our previous experiments indicated that many of the genes *activated *by Myc are normally repressed by Pc [18]. We were curious whether Pho repression overlapped Myc activation to the same extent. The levels of 680 transcripts increase with ectopic Myc. Of the 329 genes whose levels increase with *pho *RNAi, 260 are among the 680 Myc activation targets (Table 3, Figure 3E). Therefore, a substantial role for Pho in normal embryos is to repress genes that can be activated by ectopic Myc.

We examined our embryonic Myc activation targets for the presence of transcription factor binding sites. Using P-Match to search our sequences for known transcription factor binding sites within the Transfac database [41], we found 0 of 680 Myc induced targets that have canonical vertebrate Myc-binding E-boxes from the 0 position through the first exon. E-boxes in Drosophila are most likely to be found from -50 to +50, with the majority between 0 and 50 [42]. The lack of E-boxes may simply reflect the nature of our embryonic Myc targets, which exclude those that are maternally deposited because those transcripts are present in both our experimental and control embryos. It is possible that our zygotic Myc targets are part of a different set that are induced by non-canonical binding sites. Among Drosophila genes bound by dam-Myc, 95% of the sites identified at those loci were not the canonical CACGTG [43]. We did find, however, many Myc activated transcripts with vertebrate YY1 sites, which is the homolog of Drosophila Pho. The mammalian transcription factor YY1 is able to bind to PREs and recruit Pc repression in Drosophila [44], and 32 of 680 Myc activated genes have YY1 sites. To determine the significance of this number, we used P-Match to search the sequences of 50 random Drosophila genes, and 3 of those have YY1 sites as well. Therefore, we considered the appearance of 32 YY1 sites in our set of 680 Myc activated genes to be similar to what is found randomly.

### Mutations in *ash1 *cause increased expression of the *dmyc *gene

To search for other components that might mediate a Pho/Myc/Pc interaction, we conducted an unbiased genetic screen of large deletions on the Second and Third Chromosomes (comprising 80% of the *Drosophila *genome). Because *dmyc*^*BG*02383 ^flies have a darker eye color that is easier to score than *dmyc*^*BG*00605^, we used *dmyc*^*BG*02383 ^in our screen. Out of a total of 154 Deficiency mutants (Dfs), 16 scored as decreasing *mini-white *levels of *dmyc*^*BG*02383 ^and 15 scored as increasing *mini-white *expression (screen scheme is shown in Figure 1C). Among the latter Dfs, we were particularly interested in the overlapping region of Df(3L)kt02 and Df(3L)XS533 because it includes *ash1*, a histone methyltransferase [45,46]. We tested specific *ash1 *mutants for their ability to recapitulate the phenotype seen in the two overlapping deficiencies and found that 69% of *ash1*^*RE*418 ^mutant males had darker *mini-white *compared to 8% of their non-mutant siblings (Figure 2C and Table 1). To be sure that the mutations that scored as affecting the insertion in *dmyc *were indeed affecting *dmyc *expression and not *mini-white *in general, we conducted independent control crosses of a *mini-white *P element insertion (pGT1) on the X chromosome, *BG00979*, to every mutation and deficiency that scored positive in our assay. We found that 2 deficiencies affected this non-*dmyc mini-white *insertion and ruled them out. The rest, including *Pc*, *Psc*,*pho *and *ash1*, did not affect *mini-white *levels of the non-*dmyc *associated insertion (data not shown). Therefore, our genetic assay provided a DNA binding protein (Pho) and a histone methyltransferase (Ash1) as potential mediators of Myc-Pc activity.

### Depletion of Ash1 and Pc in the embryo results in similar gene expression changes

Although Ash1 appears to be involved in repression of *dmyc *with Pho and Pc in our *mini-white *assay, it is known as a Trithorax Group gene. Ash1 mutant phenotypes and genetic interactions suggest that, at least with respect to homeotic loci, it works in opposition to the activity of the Pc Group [47-51]. To examine the full range of functions for Ash1, beyond regulation of the homeotic selector genes, we conducted microarray experiments comparing gene expression changes between embryos with wild type and reduced levels of *ash1*. We injected embryos with dsRNA directed at *ash1*, reducing transcript levels 2-fold (Figure 3A). We also induced RNAi for *ash1 *in embryos with ectopic Myc to examine the role that Ash1 has in Myc function. The experiments were conducted the same way as described above for *pho *RNAi.

**Figure 6:**
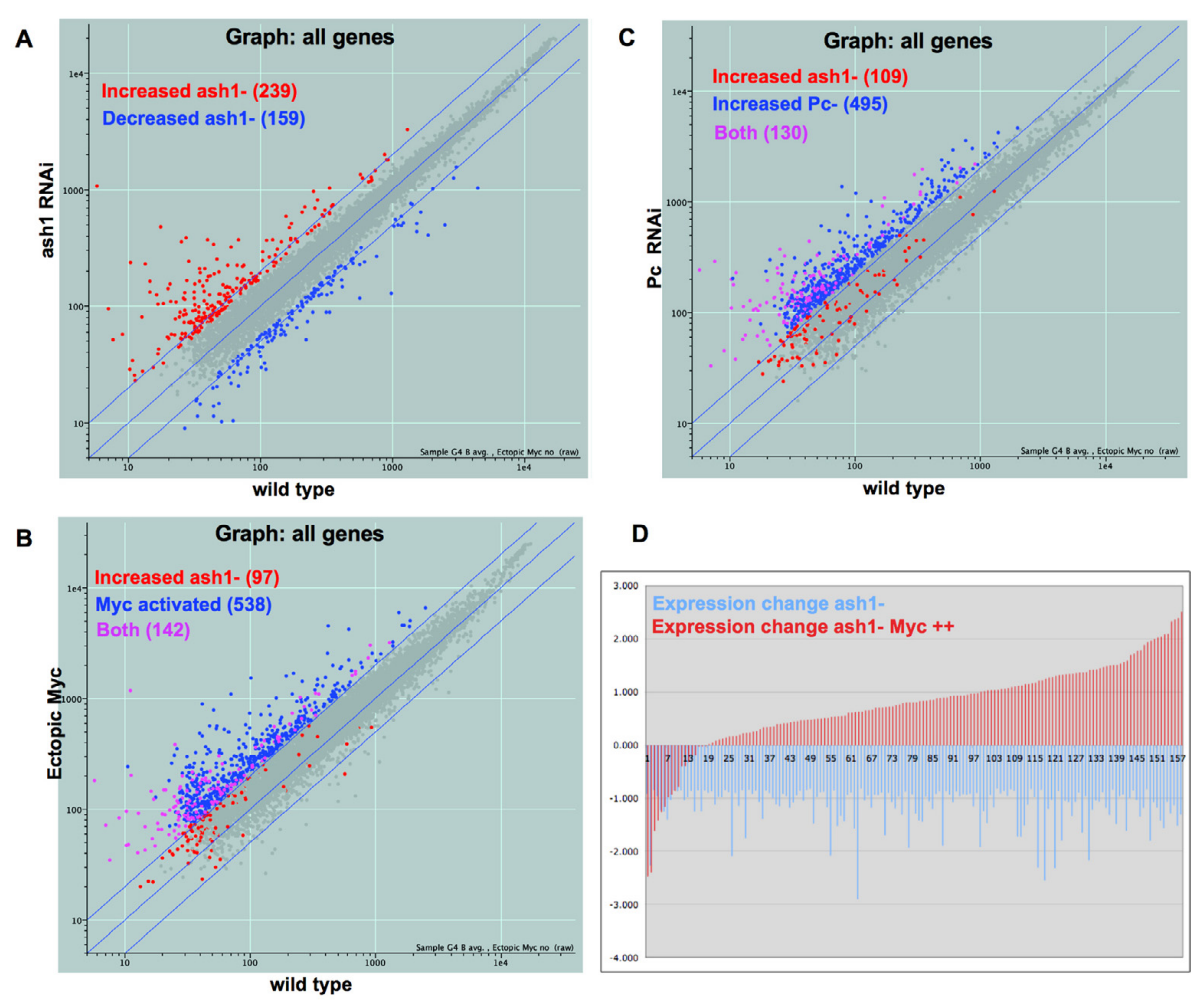
**Ash1 protein's activities overlap those of Pc and Myc**. (A) Expression levels of 8865 genes are plotted by their basal levels along the X axis and their levels upon *ash1 *RNAi along the Y axis. Red dots indicate genes whose levels rise with *ash1 *RNAi, and blue dots indicate genes whose levels decrease with *ash1 *RNAi. (B) Expression levels of 8865 genes are plotted by their response to ectopic Myc. Basal levels are along the X axis, and levels with ectopic Myc are along the Y axis. Genes in red are elevated by *ash1 *RNAi alone. Genes in blue are elevated by ectopic Myc alone, and genes in purple are elevated by both conditions individually. (C) Expression levels of 8865 genes are plotted by their response to *Pc *RNAi, plotted by their basal levels along the X axis and their levels with *Pc *RNAi along the Y axis. Genes in red are elevated by *ash1 *RNAi alone, genes in blue are elevated by *Pc *RNAi alone, and genes in purple are elevated by both conditions individually. (D) Ectopic Myc replaces Ash1 in gene activation. Changes in expression of 160 genes are shown, log_2 _of ratios of expression over basal, for transcripts whose levels drop with *ash1 *RNAi (blue bars). For each of the 169 genes, the change in expression in embryos with *ash1 *RNAi combined with ectopic Myc is also shown (red bars, next to blue bar for each gene).

We first characterized Ash1's potential repressive activity in the embryo, determining whether *ash1 *RNAi causes increased expression of certain transcripts and whether those targets overlap Myc or Pc activities. We found that levels of 239 genes increase with *ash1 *RNAi, suggesting that Ash1 behaves directly or indirectly as a repressor in the embryo (Table 2, Figure 6A). 142 of these 239 genes are activated by Myc (Figure 6B, purple). The overlap parallels our previous findings with Pc and Pho, and suggests that, in the embryo, a significant role of Ash1, Pho and Pc is to control the expression of targets that are activated by ectopic Myc. In addition, we found that 130 of the 239 Ash1 repression targets are also Pc repression targets (Figure 6C, purple). This overlap is at least as large as that observed for Pho and Pc and provides the first molecular link between Pc and a TrxG protein in repression. This link may be the result of either direct or indirect activities of Pc and Ash1. The overlap of Pc/Pho and Ash1 repression targets is not limited to genes activated by Myc. 66 of the 97 non-Myc targets repressed by Ash1 are also repressed by Pc or Pho.

The genetic assay that revealed that Ash1 is involved in Myc biology was one assessing repression of the *dmyc *locus. Therefore, we examined the 57 Myc repressed genes for any requirement of Ash1 in that repression. In an embryo with ectopic Myc and *ash1 *RNAi, 47 of those genes remain repressed (Table 3) suggesting that Ash1 is required for Myc repression at only a small fraction of its targets (i. e., 10/57 = 18%). In contrast, 25 of the same 57 Myc repressed targets were no longer repressed to the same degree when *Pc *levels were reduced. This effect is more pronounced than that of Ash1 and may indicate a more direct role for Pc in Myc repression. These numbers do not represent absolute repression by Ash1 and Pc, however, given that the RNAi depletion of these transcripts was not complete. In fact, *Pc *levels were reduced 2 fold more than *ash1 *transcripts, as measured by microarray.

### Ectopic Myc replaces Ash1 in activation

Consistent with Ash1's known role in gene activation, levels of 159 genes decrease in response to *ash1 *RNAi (Figure 6A, Table 2), an effect that is superficially similar to the positive role of Ash1 in regulating homeotic gene activity. 70 of these 159 genes are also Myc activation targets. In contrast, only 9 of 76 genes whose levels decrease with *Pc *RNAi are also Myc activated (Table 3). Similarly, just 4 of 69 genes whose levels decrease with *pho *RNAi are also Myc activated (Table 3), suggesting that Pc and Pho are unlikely to be functioning similarly to Ash1 with respect to gene activation. Given this overlap between activation targets of Myc and Ash1, we hypothesized that ectopic Myc might rescue the loss of activation that occurs with *ash1 *RNAi. If this were the case, we would expect that genes whose levels decrease with *ash1 *RNAi alone would in fact increase with *ash1 *RNAi and ectopic Myc. Consistent with this notion, we found that levels of 81 of these 159 genes are significantly elevated above basal levels in embryos with both ectopic Myc and *ash1 *RNAi, and expression of 68 genes is at basal levels (Figure 6D). Only 10 of the 159 genes whose levels are reduced with *ash1 *RNAi remain reduced in the presence of ectopic Myc. Therefore, a large majority of genes that are activated by Ash1 are not only Myc activated, but Myc can replace Ash1 in their activation. These results also suggest that, while Ash1 is important in the activation of these genes, they do not require wild type levels of Ash1 to be activated by Myc.

### Ash1 is required for Myc activation

**Figure 7:**
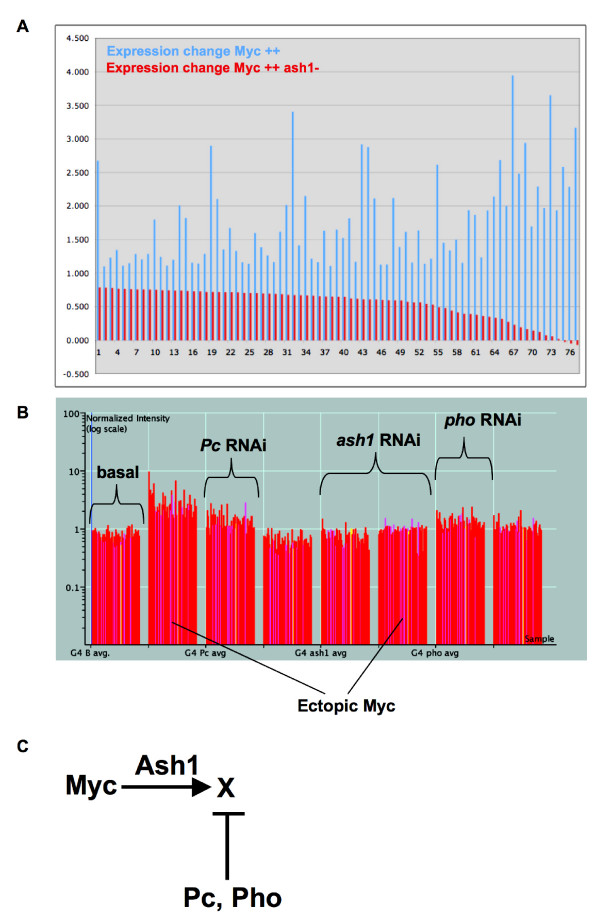
**Ash1 works against Pc/Pho repression to facilitate Myc activation**. (A) Changes in expression of 77 genes are shown, log_2 _of ratios of expression over basal, for transcripts whose levels increase with ectopic Myc (blue bars). For each of the 77 genes, changes in expression are also shown in embryos with ectopic Myc and *ash1 *RNAi (red bars, next to the blue bar for each gene). (B) A graph showing the expression of 60 genes in response to eight genetic conditions, as clustered by K-means. The log_10 _scale of normalized expression for each gene is represented as a vertical line, and along the X axis left to right, the 60 are grouped by sample: *Gal4 *alone, *Gal4 *Myc, *Gal4 Pc *RNAi, *Gal4 *Myc *Pc *RNAi, *Gal4 ash1 *RNAi, *Gal4 *Myc *ash1 *RNAi, *Gal4 pho *RNAi, *Gal4 *Myc *pho *RNAi. These Myc activated genes fail to be activated with *ash1 *RNAi (compare 2^nd ^and 6^th ^sets), and they are also repressed by Pc and Pho (compare 1^st^, 3^rd ^and 7^th ^sets). (C) A Model suggesting a possible regulation scheme for the genes shown in B, "X".

We were curious about the inverse, whether Ash1 is required for activation by Myc. Levels of 680 genes increase upon activation of ectopic Myc. In embryos with both ectopic Myc and *ash1 *RNAi, levels of 76 of these genes fail to increase at the same level (Figure 7A). None of these 76 genes is also regulated by Ash1 alone, suggesting that Ash1's regulation of these genes is limited to activation by Myc. Upon closer examination of the 76 genes, we found a cluster of 60 that are regulated by Pc and Pho (Figure 7B, generated by K-means clustering of Myc activation targets). The regulation of these genes by Pc and Pho is negative, in that the targets have elevated expression with *Pc *or *Pho *RNAi (Figure 7B). Therefore, many of these targets are normally repressed by Pc/Pho. Among the named genes in this group, several encode structural constituents, such as Ccp84Ac (larval cuticle), Dhc62B (Dynein Heavy chain 62B) and Actin 88F, and some are involved in metabolism, such as Glycogenin and Lipase1. One possible explanation for the regulation of these Myc targets is that the role of Ash1 is to oppose Pc/Pho repression, allowing Myc activation. In fact, Ash1 opposes Pc repression at homeotic loci [47]. Therefore, our data suggest that Ash1 also opposes Pc repression at loci activated by Myc (Figure 7C).

### Myc targets exhibit disparate histone modifications

**Figure 8:**
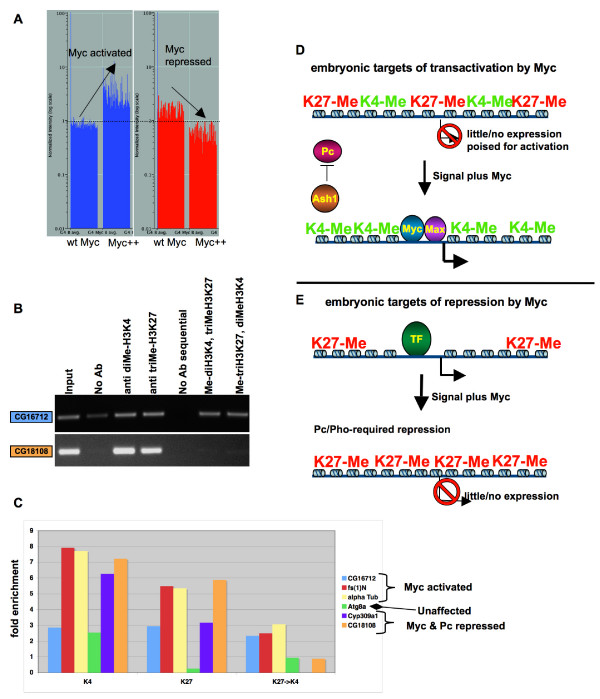
**Myc targets of activation are methylated at both H3K4 and H3K27**. (A) (Left) A graph showing basal levels of 680 Myc activated targets (blue, left bar, each gene is represented by a thin line) followed by their levels upon ectopic Myc activation (blue, right bar, same genes as in the left bar). (Right) Basal levels of 57 Myc repression targets are shown (red, left bar, each gene is represented by a single thin line), and their levels following the activation of ectopic Myc (red, right bar, same genes as in the left bar). Compare basal levels of the activation targets versus repression targets. (B) Myc activation targets that are also regulated by Pc, Pho and Ash1 are methylated at both H3K4 and H3K27. DNA purified from chromatin immunoprecipitation reactions provided the template for PCR of *CG16712 *(upper) and *CG18108 *(lower), and the ChIP antibodies used are indicated above each lane. The right two lanes show results from sequential ChIP of chromatin by one antibody and then another. Input chromatin DNA is shown the far left lane, used in a 1:1000 dilution. (C) The enrichment by IP of chromatin containing 6 genes was calculated by dividing the levels of PCR product by that of the background, no antibody control. *CG16712*, *aTub67C *and *fs(1)N *are all Myc induced, Pc, Ash1 and Pho repressed, and show methylation of H3K4 and K3K27 together. *Atg8a *is not affected by any of the four regulators, though it is highly expressed in the embryo and methylated at H3K4 (blue bar). *Cyp309a1 *and *CG18108 *are Myc/Pc repressed, and show both H3K4 and H3K27 methylation in wild type embryos, but not at the same locus by sequential ChIP. (D) A target of activation by Myc is depicted, with low levels of expression, in a domain of chromatin bearing H3K27 methylation and H3K4 methylation. A growth signal or other signal, including increased Myc accumulation, allows Myc/Max to bind to a binding site, recruiting activators and inducing transcription. Ash1 is shown directly or indirectly repelling PcG repression and maintaining an active H3K4 methylation state. (E) A target of Myc repression is depicted, with high levels of expression mediated by an unknown transcription factor (TF). A cellular signal and increased Myc accumulation allow Pho and Pc required repression, propagating a repressed chromatin state characterized by H3K27 methylation.

In many of our experiments, Pc, Pho and Ash1 proteins facilitate Myc's activity as a repressor, but also appear to oppose its activity as an activator. In an attempt to understand how the same proteins cooperate with Myc to repress one set of targets and conversely work to repress a different set of Myc activation targets, we have to consider a major difference between Myc's targets of activation and repression: before Myc acts upon them, repression targets are necessarily being expressed while activation targets are largely unexpressed (Figure 8A).

Myc's embryonic targets of activation, prior to induction of ectopic Myc, are expressed at low levels and our data show that Pc, Pho and Ash1 are required to maintain that pattern of expression. To examine Myc targets for hallmarks of both Ash1 and Pc activity, we used chromatin immunoprecipitation to examine the methylation of histone tails in wild type embryos. Because Ash1 methylates H3K4, a chromatin modification that correlates with mammalian targets of Myc activation [6], and Pho and Pc are involved in setting up and binding to H3K27 methylation, respectively, we used sequential chromatin immunoprecipitation to determine whether Myc activation targets in the embryo exhibit both methylation marks. Using chromatin from wild type embryos, obtained over a 24 hour room temperature collection, we immunoprecipitated with anti-dimethyl H3K4, purified the chromatin, and immunoprecipitated again with anti-trimethyl H3K27. We also performed the reciprocal experiment, beginning with anti-trimethyl H3K27 and following with anti-dimethyl H3K4 (See Methods, and [52]). We used PCR to detect three different genes that are Myc activated, Pc, Ash1 and Pho repressed (*CG16712*, *fs(1)N*, and *alpha Tubulin 67C*, Figure 8B–C). All three of these genes were amplified from our sequential ChIP DNA, arguing that they are modified on both H3K4 and H3K27. As a control, we examined a gene that is not regulated by Myc, Pc, Ash1 or Pho (*Atg8a*) and found it absent in our sequential ChIP DNA, though it is expressed at high levels in the embryo and methylated at H3K4 (Figure 8C). In addition, we tested two genes that are Pc and Myc repressed (*CyP309a1*, *CG18108*, respectively [18]), and they show both H3K4 and H3K27 methylation individually in chromatin from wild type embryos. This result is not unexpected, since we obtained the chromatin from all the cells of wild type embryos, so that different cells may be expressing or repressing each gene. We found that these loci are not methylated at both residues simultaneously, because we failed to amplify either gene from our sequential ChIP DNA (Figure 8B–C). These results, along with our genetic and genomic data, support a model for the control of Myc targets in the embryo, in which Ash1, Pc and Pho are required for the low levels of expression of these genes, reflected by the disparate chromatin methylation of targets of Myc activation (Figure 8D). Developmentally regulated genes in mammalian embryonic stem cells also exhibit low levels of expression prior to differentiation, corresponding to methylation of both H3K4 and H3K27 [52].

Myc repression targets, different from activation targets, are expressed in the embryo prior to ectopic Myc induction (Figure 8A). Bracken and colleagues have shown that PcG proteins bind at expressed loci prior to differentiation and repression, and those sites also have H3K27 methylation [22]. We have observed H3K27 methylation of Myc targets in wild type embryos (Figure 8B–C), suggesting that they are primed for repression, and upon ectopic Myc induction Pho and Pc work to repress these targets. The role of Ash1 in repression of these targets may be limited to the embryo, possibly to allow for trans-activation in the event of an activation signal, rather than a less reversible Pc-mediated silenced state (Figure 8E).

## Discussion

### The requirement for Ash1 in activation and maintenance of non-activation

Our genetic assay for modification of expression of the *dmyc *locus enabled us to identify an unexpected interaction, namely that gene regulation by Ash1 in the embryo overlaps gene regulation by Myc, Pc and Pho. We found that a deletion of *ash1 *leads to greater expression of a *mini-white *reporter within the *dmyc *locus, which potentially reflects repression of *dmyc *in the eye imaginal disc. In addition, *ash1 *RNAi in the embryo causes the induction of 239 genes. Interestingly, homeotic transformations in flies mutant for *ash1 *are enhanced by mutations in *Psc *[53], a PcG gene that is part of the core repressive complex including Pc [34]. Thus, the current understanding of Ash1 as a nuclear protein involved in the complex regulation of cellular memory does not exclude the possibility that Ash1 may be involved in repression of certain genes, directly or indirectly. Ash1 was identified as a late larval lethal mutation with imaginal disc defects [54]. Expression of homeotic selector genes in *ash1 *mutants is affected differently in different cells, with stochastic loss of expression [48] apparently caused by ectopic silencing by Pc-G proteins [47]. Flies heterozygous for a mutation in *ash1 *show homeotic transformations when the fly is also heterozygous for another *trxG *mutation [51]. This intergenic non-complementation indicates that levels of TrxG proteins are important, which is supported by the large genomic effects we describe with just a 2-fold reduction in *ash1 *transcripts. The Ash1 protein is a SET domain histone methyltransferase that methylates lysines 4 and 9 of histone H3 and lysine 20 of histone H4, and these combined activities are correlated with active transcription [45,46]. Interestingly, methylation of histone H3 lysine 9 is reduced in the chromocenter of polytene chromosomes in *ash1 *mutants lacking the SET domain [46], and H3K9 methylation is associated with HP-1 binding and heterochromatin formation [55-57]. In addition, Ash1 binds to 108 bands on polytene chromosomes, several of which overlap or are adjacent to binding sites of Psc [50]. We found that among the 239 genes that are up-regulated with *ash1 *RNAi, 130 are also up-regulated with *Pc *RNAi, 139 are up-regulated with *pho *RNAi, with 73 of these genes common to all three. These results suggest that Ash1 methylation is not exclusive to transcriptional activation.

Ash1 also appears to be involved in activation of a different set of embryonic targets, and many of the genes in this set overlap Myc activation targets. In fact, we found that ectopic Myc can replace Ash1 in embryos with both *ash1 *RNAi and ectopic Myc by activating 76% of the genes whose expression decreases with *ash1 *RNAi alone. In addition, we argue that Ash1 is required in Myc activation of a subset of its targets, presumably by antagonizing Pc/Pho repression. H3K4 methylation is correlated with Myc binding to high affinity target sites, and this methylation occurs in the absence of Myc protein, suggesting that it is established and then Myc is able to bind [6]. Ash1 may function to maintain a favorable methylation state of Myc targets, allowing subsequent activation by Myc.

We found minimal direct overlap of Ash1 activation with Pc and Pho repression. Our threshold values for determining genes affected by each genetic condition likely caused false negative results; in addition, *ash1 *transcript levels were not depleted as effectively as *Pc *or *pho *levels. Our experiments were conducted with RNA from entire embryos, therefore we cannot determine the segment-specific regulation by the genes that we tested, and the overlap of Pc and Pho repression with Ash1 activation would be expected to occur in a segment-specific manner. Following that argument, the large overlap of Ash1, Pc and Pho *repression *targets may be related to universal genetic programs, including Myc transcriptional activation, in contrast to the cell-type specific programs, such as those under the control of homeotic genes.

### Pho's activity overlaps Myc targets better than Pc targets

We found many of the transcripts that are de-repressed by *pho *RNAi overlap those that are de-repressed by *Pc *RNAi, but more of the Pho targets overlap those that are Myc activated than Pc repressed. Our stringent statistical requirement for the membership of any transcript in a list of genes that increase or decrease in response to a particular genetic condition may reduce our view of the overlap of genes regulated by both Pc and Pho. But a difference in the effects of loss of Pc and loss of Pho is not unprecedented. *pho *mutants have segmental transformations similar to those of *Pc *mutants, however these mutations have either little or no effect on expression of *abd*-*A*, *abd*-*B *or *Ubx *in embryos [20,58-60]. And yet, Pho is required for silencing of the iab-7 PRE [61] and is necessary, though not sufficient, for the silencing activity of the MCP element of the Abd-B gene [62]. Our data and the earlier experiments we described may reveal the redundancy of Pho and Pho-like, which is a homolog of Pho and binds to the same sequence [28,63]. Wang and colleagues found that Pho-like recruits PcG complexes just as Pho does in the wing imaginal disc [32].

## Conclusion

Our data have revealed that there is tremendous overlap and plasticity of regulation by proteins whose functions have been thought to be disparate. We are left with a view of the embryonic genome in which the transcription factor Myc is capable of activation and repression of many loci, and its canonical binding site is less important than the influence of proteins whose role in biology is to maintain cell fates. The methylation status of chromatin surrounding Myc targets supports the notion that the nucleus of a wild type embryo is primed to control its response to a transcription factor such as Myc.

## Methods

### Fly strains and crosses

See Flybase for additional information about the mutants and insertions used. *dmyc*^*BG*02383^, *dmyc*^*BG*00605^, and BG00979 were generated by the *Drosophila *P-Screen/Gene Disruption Project.

For the RT-PCR data shown in Figure 1, we crossed females homozygous for *dmyc*^*BG*02383 ^on the X Chromosome and homozygous for a maternal *tubulin Gal4 *driver on the Second and Third chromosomes (*matTub-Gal4VP16 67C;15*) to males hemizygous for *dmyc*^*BG*02383 ^on the X Chromosome and homozygous for *UAS-dmyc *on the Third chromosome [64]. For control embryos lacking ectopic Myc, we crossed *dmyc*^*BG*02383 ^; Gal4 driver females to their siblings. For ectopic expression of *dmyc *used in the microarray experiments, females homozygous for an *armadillo-Gal4 *driver [65] were crossed to males homozygous for *UAS-dmyc *[64]. Control embryos were collected from mothers and fathers homozygous for *armadillo-Gal4*.

For the *mini-white *assay for *dmyc *expression, we crossed females homozygous for each insertion on the X Chromosome to males heterozygous for *Pc*, *Psc*, *E(z) *and *pho *mutations and scored all the male progeny by their eye color (all the male progeny of the cross will be hemizygous for their mother's X chromosome, *dmyc*^*BG*02383 ^or *dmyc*^*BG*00605^, and heterozygous for either a mutant chromosome or the balancer). We scored the males within a few hours of eclosing and grouped them based on intensity of *mini-white *expression. We counted the flies in each of three groups (light, dark, neither), and looked for differences in representation of males with the mutation versus males with the balancer chromosome (effectively wild type). We crossed *dmyc*^*BG*02383 ^homozygous females to males of the Bloomington stock center's deficiency collection that includes deletions spanning 80% of the Second and Third Chromosomes (Figure 1C). We scored F1 males as described for *Pc*, *Psc*, *E(z) *and *pho *mutations.

### RNAi

We amplified 500 bp of 5' regions of the *ash1 *and *pho *cDNAs, and cloned the products using the pGem T Easy vector (Promega). We synthesized the sense and antisense transcripts separately using the dual promoters of pGem T Easy, SP6 and T7 polymerases (Ambion), and annealed the transcripts to each other. We injected a 5 μM solution of the dsRNA into dechorionated embryos, as previously described [66]. Injected embryos were washed off the cover slip with heptane to solubilize the glue and oil. We removed the heptane and froze the embryos before homogenization in TRIzol.

Injected embryos were all alive and undergoing germ band retraction (approximately mid-embryogenesis) before RNA isolation. None exhibited any mutant phenotype, including those with ectopic Myc, which is lethal by the end of embryogenesis.

### RNA isolation and RT-PCR

We used TRIzol reagent (Invitrogen) to isolate total RNA from dechorionated embryos. RT-PCR reactions were performed using Invitrogen's SuperScript One Step RT-PCR system, as previously described [18]. Primer pairs for all amplification products spanned an intron to control for DNA contamination. We minimized amplification cycles in order to remain in the linear range of amplification.

### Microarray data treatment

We began our analysis of the data by eliminating genes from our analysis whose levels were below a threshold. We determined that threshold using signals for negative controls on the arrays, such that genes whose levels were less than 2.5 times the signal for a negative control were not considered in our analysis. In the next step we normalized the raw data by averaging the signals of several *Drosophila *positive controls (4 different actin signals), comparing those averages across arrays (16 arrays, from biological replication of 8 samples), and multiplying all signals by a resulting chip-specific constant. We then averaged signals for each gene in biological replicates. We were left with data for eight experiments, normalized to be comparable.

To compare changes in gene expression between genetic conditions, we generated ratios of expression for each genetic condition by dividing all signals by their corresponding signal in the *Gal4 *no RNAi sample. As described previously, we took the log_2 _for each ratio, found the mean of the ratios for each of seven lists of ratios, determined the standard deviation from the mean, and classified genes as changing up or down based on the standard deviation from the mean. Genes whose ratios were 1.5 times the standard deviation were classified as being induced or de-repressed, and genes whose ratios were -1.5 times the standard deviation were classified as being repressed or de-activated (in the cases of up with *Pc*- and Myc activated, we used 2 times the standard deviation, because levels for many more genes went up than down in those samples). Following this classification, we established lists for genes as follows: up with Myc, down with Myc, up with *ash1*-, down with *ash1*-, up with *pho*-, down with *pho*-, up with *Pc*-, down with *Pc*-, up with Myc *ash1*-, down with Myc *ash1*-, up with Myc *pho*-, down with Myc *pho*-, up with Myc *Pc*-, down with Myc *Pc*-. We generated these lists using normalized data in Excel, and loaded the raw data and our lists into GeneSpring for clustering and figure generation. Our complete data set is available in Additional file 1. Cel files and other file types containing the data are available upon request.

### Chromatin immunoprecipitation

We performed single ChIPs as described previously [18], and sequential ChIPs as described by Bernstein and colleagues [52]. We used anti tri-methyl H3K27 (Upstate) at a 1:100 dilution, and anti di-methyl H3K4 (Upstate) at a 1:100 dilution, and used primers to amplify the 5'-most 400 bp of each gene. For single ChIPs we used 30 amplification cylces, and for sequential ChIP we used 35 cycles. Band intensities of PCR products were determined using an Alpha Innotec device and Fluor-Chem software.

## Abbreviations

Pc = Polycomb

pho = pleiohomeotic

ash1 = absent, small or homeotic discs 1

*dmyc *= *Drosophila *myc gene; diminutive

dMyc = *Drosophila *Myc protein

RNAi = RNA interference

PcG = Polycomb Group

TrxG = Trithorax Group

PRE = Polycomb Response Element

PRC1 &2 = Polycomb Repressive Complexes 1&2

Df/Dfs = Deficiency/Deficiencies

H3K27 = histone H3 lysine 27

H3K9 = histone H3 lysine 9

## Authors' contributions

JMG designed and performed experiments, analyzed data, and prepared the manuscript. EW and MDC assisted in experimental design and data interpretation, edited the manuscript, and provided funding. All authors have read and approve the final manuscript.

## Supplementary Material

Additional file 1 Microarray data. A tab-delimited txt file containing the Affymetrix intensities of our averaged, replicated chip hybridizations. Click here for file
